# Lazy workers are necessary for long-term sustainability in insect societies

**DOI:** 10.1038/srep20846

**Published:** 2016-02-16

**Authors:** Eisuke Hasegawa, Yasunori Ishii, Koichiro Tada, Kazuya Kobayashi, Jin Yoshimura

**Affiliations:** 1 Laboratory of Animal Ecology, Department of Ecology and Systematics, Graduate School of Agriculture, Hokkaido University, Sapporo 060-8589, Japan.; 2 Graduate School of Science and Technology and Department of Mathematical and Systems Engineering, Shizuoka University, 3-5-1 Johoku, Naka-ku, Hamamatsu 432-8561, Japan; 3 Marine Biosystems Research Center, Chiba University, Uchiura, Kamogawa, Chiba 299-5502, Japan; 4 Department of Environmental and Forest Biology, State University of New York College of Environmental Science and Forestry, Syracuse, NY13210 USA

## Abstract

Optimality theory predicts the maximization of productivity in social insect colonies, but many inactive workers are found in ant colonies. Indeed, the low short-term productivity of ant colonies is often the consequence of high variation among workers in the threshold to respond to task-related stimuli. Why is such an inefficient strategy among colonies maintained by natural selection? Here, we show that inactive workers are necessary for the long-term sustainability of a colony. Our simulation shows that colonies with variable thresholds persist longer than those with invariable thresholds because inactive workers perform the critical function of replacing active workers when they become fatigued. Evidence of the replacement of active workers by inactive workers has been found in ant colonies. Thus, the presence of inactive workers increases the long-term persistence of the colony at the expense of decreasing short-term productivity. Inactive workers may represent a bet-hedging strategy in response to environmental stochasticity.

Optimality theory predicts the maximization of individual fitness^1^,^2^,^3^ (also 7–9 pp. in ref. 2; 5,6 pp. in ref. 3). This theory also predicts an increase in the average short-term productivity of a functional unit because such a change will usually increase the fitness of all members^4^ (see also 119–129 pp. in ref. 5). A colony of social insects is a functional unit subjected to natural selection for high productivity^5^,^6^. Such selection has been demonstrated empirically in the behavioral caste ratio in *Pristomyrmex punctatus*^7^ and the morphological caste ratio in *Camponotus nipponicus*^8^. However, various studies that have estimated the ratio of inactive workers in a colony to be approximately 50% of workers on average^9^,^10^,^11^,^12^,^13^,^14^,^15^,^16^,^17^,^18^,^19^,^20^,^21^, even in wild colonies^22^. Furthermore, many workers continue to remain inactive for long periods^21^,^23^,^24^. Although the short-term productivity is maximized at the colony level, the existence of so many inactive workers has been a focus of recent behavioral studies of social insects^20^,^21^, because these conditions seem to be far from optimal for the maximization of short-term colony productivity.

How are such inactive workers generated in a colony? Individual workers in social insect colonies vary in their response thresholds for task stimuli^25^,^26^,^27^. An individual with high thresholds for all tasks will tend to be inactive because most tasks exert an insufficient task-related stimulus to induce work. Thus, workers with high thresholds for every task fail to work for most tasks encountered^24^. Only when there are no other workers around will an inactive worker proceed with a task, because the task stimulus continues to increase when it is not processed by other workers. Observational studies have shown that when more than half of the workers have processed less than 20% of the social tasks, as indicated by their behaviors, inactive workers start to work only when the active workers had been removed from the colony^24^. Thus, this evolutionary strategy results in considerably low colony efficiencies compared with the potential efficiency that could be realized if all workers were to work simultaneously.

Why have insect societies adopted such an inefficient strategy from the perspective of maximizing short-term productivity? All animals fatigue after processing a series of tasks and need to rest to recover. Therefore, inactive workers should benefit the colony by replacing active workers when they become fatigued and need to rest for recovery. In the present study, we investigated the effects of fatigue on the labor dynamics of insect societies.

In insect colonies, some tasks are considered to be so critical that they must be performed continuously (e.g., egg cleaning and larval care). Indeed, the eggs of termites are known to die from bacterial infections if their cleaning is halted even for very short periods^28^. Once a catastrophic disaster happens, all active workers are to become completely fatigued simultaneously, so that no tasks can be processed by these active workers. If there is no inactive workers, no worker could engage in such crucial tasks, resulting in serious damage to the survival of the colony. However, if there are sufficient numbers of inactive workers with very high thresholds for task stimuli, they are capable of performing those crucial tasks when all active workers become completely fatigued due to the disaster. Then, the colony is effectively buffered from experiencing such dangerous conditions. Thus, colonies with a high proportion of inactive workers are likely to persist considerably longer than those without such a “failsafe” system, even though the short-term productivity of such colonies becomes much lower than those with no inactive workers (or constant/invariable thresholds).

The above problem indicates that a colony always faces with the tradeoff between long-term persistence and short-term productivity. In this study, we test that inactive workers are necessary for the long-term sustainability of insect societies. We hypothesize that inactive workers act as a safeguard (reserve) against rare catastrophic disasters, when all active workers must take a break simultaneously due to the fatigues resulting in the disasters. If this hypothesis is correct, the colonies with variable thresholds for task stimuli will persist longer than those with constant threshold. We built a simulation model of the labor dynamics of an insect colony system with variable thresholds for task stimuli and compared this to a colony without variable thresholds as a control. We show that the persistence of colonies with variable thresholds is longer than that of colonies with invariable thresholds. We also examined whether social task replacements took place between active and inactive workers in these simulation runs. We then empirically examined ant colonies with inactive workers to detect social task replacements in these colonies. We finally discuss the proposed theory and how this theory is theoretically equivalent to the concept of geometric mean fitness in stochastic environments.

## Results

We conducted a lattice-model simulation of 2500 (=50 × 50) cells with 75 workers. A task of a certain stimulus (5.001 stimulus) appears randomly with a given appearance rate and increases its stimulus value by one unit when it is not processed by a worker. A worker moves randomly to a neighboring cell, and when a worker encounters a task, it will process the task if its stimulus value is higher than the worker’s threshold. In the system with variable thresholds, the threshold value of a worker is assigned following a normal distribution (ranging between 0–10 with mean =5). In the system with invariable thresholds, all workers are assigned the threshold of 5 (control experiment). We introduce the fatigue measure of a worker as a power level as follows. Initially, the power level of all workers is set to 10. When a worker processes a task, its power level is reduced to 0 (zero) and the worker is immobilized (no movement and no task processing). At each time step, the worker recovers from the fatigue at a given constant rate. We examine the effects of fatigue in the colony system by altering the recovery rate and task appearance rate.

**Figure 1:**
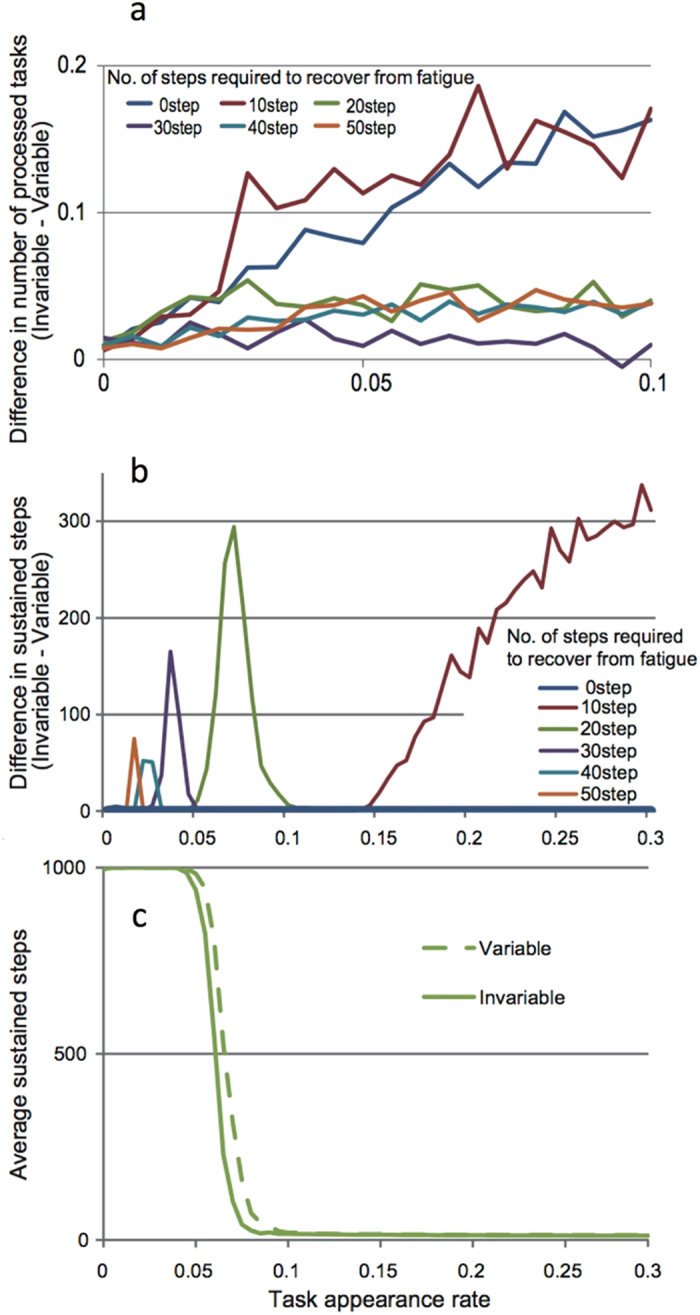
Sustainability of a colony with/without inactive workers. (**a**) The number of processed tasks in a time step is always larger in a colony with an invariable response threshold among workers irrespective of parameter values (degree of fatigue and task appearance rate), indicating that variable threshold systems are generally more productive in short-term. (**b**) Variable threshold systems persisted longer than systems with equivalent thresholds. The advantageous parameter space of the variable threshold system differs depending on the task appearance rate. When there is no fatigue (black line) there is no difference in the time of persistence between the two systems. (**c**) Change in sustained rate of colonies against task appearance rate. In both the variable and invariable systems, the sustained rates decreased with the task appearance rate, but the decline began earlier in the system with invariable thresholds. This difference generates convex shapes of advantageous areas of parameter space in which variable threshold systems persist for longer.

The results of all simulations show that the efficiency of the variable system is always lower than that of the invariable system. The number of tasks processed is always smaller in the variable system for every combination of task appearance and fatigue recovery rate (Fig. 1a). This consistent difference arises because there are several workers with a high threshold that do not process the tasks with a lower stimulus in the variable system. When there is no fatigue (i.e., a worker recovers in the next step), the differences in the processed tasks increase almost linearly with the task appearance rate (black line in Fig. 1a). Interestingly, differences in the processed tasks between the variable and invariable systems become smaller when the fatigue level is high (i.e., the recovery rate is low). This diminishing difference between the systems suggests that most workers become tired and immobilized irrespective of threshold variability, resulting in the reduction in the total number of tasks processed. Thus, our results clearly demonstrate that short-term efficiencies are always lower in the variable system.

We compare the persistence of a colony between the variable and invariable systems assuming that the colony becomes extinct when no task is processed during a single time step (Fig. 1b). For each recovery rate, we record the number of elapsed time steps of both variable and invariable systems along each task appearance rates. As task appearance increases, the length of colony persistence (as measured in time steps) decreases more rapidly in the invariable system than in the variable system (Fig. 1c). Figure 1b shows a plot of the differences between the two curves for various recovery rates. When fatigue is introduced (i.e., recovery rate >1), the variable system always exhibits higher persistence at several task appearance rates (colored lines in Fig. 1b). However, when there is no fatigue (recovery rate = 1), the persistence of a colony becomes identical between the variable and invariable systems (blue line in Fig. 1b). When the recovery rate is faster, the ranges become higher and wider and the differences in persistence become larger (differences in color lines in Fig. 1b).

**Figure 2:**
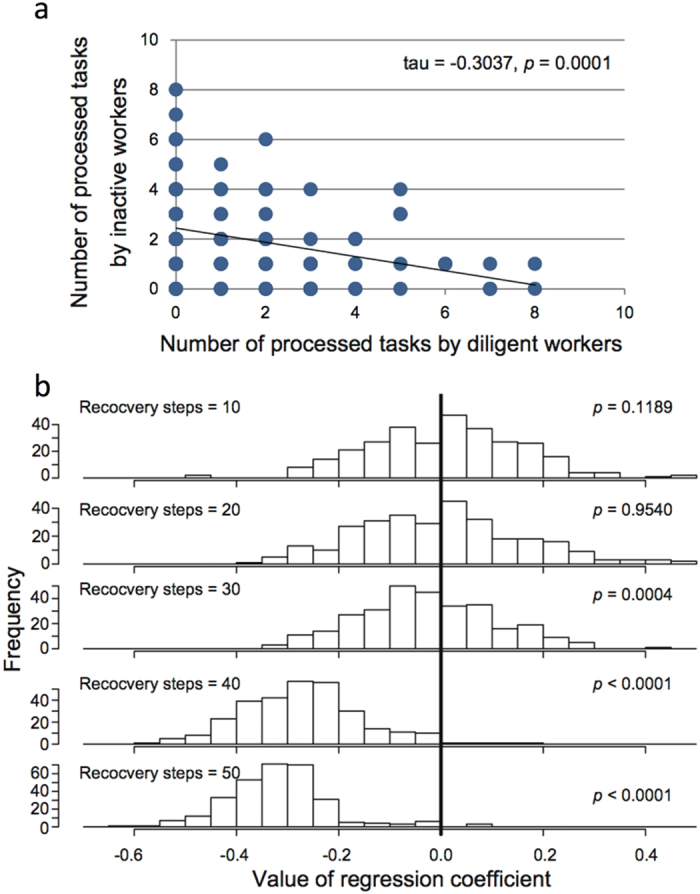
Simulated correlations in the number of processed tasks between active and inactive workers. (**a**) The correlation in the number of processed tasks in a time step between active (top 10%) and inactive (bottom 10%) workers in a representative simulation run. There is a negative relationship between active and inactive workers, meaning that inactive workers process more tasks when active workers rest. Task replacements of fatigued active workers by inactive workers explain this negative correlation. (**b**) Distributions of the above correlation among several different degrees of fatigue. When the degree of fatigue is low (tired workers recover from fatigue sooner), the distribution is positively skewed. This trend indicates that task replacements occur only under high degrees of fatigue, and that no fatigue does not require any reservoir because all workers can process tasks in the next time step (such a condition is impossible in actual animals).

The difference in colony persistence should be related to the probability of blank steps to occur when no task is processed (i.e., “extinction”). In the invariable system, all workers are likely to be tired simultaneously at some point, resulting in a blank step with a high probability. In contrast, in the variable system, inactive workers are likely to process the tasks that active but fatigued workers are incapable of processing. We therefore examined whether such task replacements by inactive workers actually take place in these simulation processes. We chose the 10% of workers with the lowest and highest thresholds and analyzed the correlation between the number of processed tasks at a time step between the two individual classes in each simulation run (an example is shown in Fig. 2a). There was a negative correlation between them, suggesting that inactive workers replaced active, but resting, workers in processing tasks. Figure 2b shows the distributions of regression coefficients by the degree of fatigue. There is no bias toward negative values under low degrees of fatigue. However, as the degree of fatigue increases, the distribution becomes positively skewed. These results suggest that a low degree of fatigue promotes a rapid recovery of active workers from fatigue; thus, almost no task replacement occurs because crucial tasks have been processed by recovered active workers. In contrast, under heavy fatigue, inactive workers begin to work because fatigued workers are not able to recover quickly. Thus, task replacements were observed only under large degrees of fatigue. In principle, the invariable system has no inactive workers available to perform such task replacement.

The current simulation results predict a negative correlation between the activity of inactive and active workers. We therefore examined the predicted negative correlation empirically in the behavioral activities of ant workers of *Myrmica kotokui*. The behaviors of the workers have been classified into 16 categories, of which 12 are recognized as social tasks^24^. We followed the previous study^24^ for the definitions and categorization of social behavior that contribute the other nest colony members. The categories of activities are allo-grooming, brood care, regurgitation and foraging. Those of non-activities are rest, walking and self-grooming. First, the existence of inactive workers was confirmed as follows. The observations of 8 colonies showed that 66.0 ± 9.8% (mean ± S.D.) of behaviors were “resting” per observation period (scan). Furthermore, 12.3 ± 11.0% (mean ± S.D.) of workers performed social tasks in less than 5% of the observation time over all scans and 26.3 ± 19.9% performed these tasks in less than 10% of the observation time. These results confirmed the existence of inactive workers in these ant colonies.

**Figure 3:**
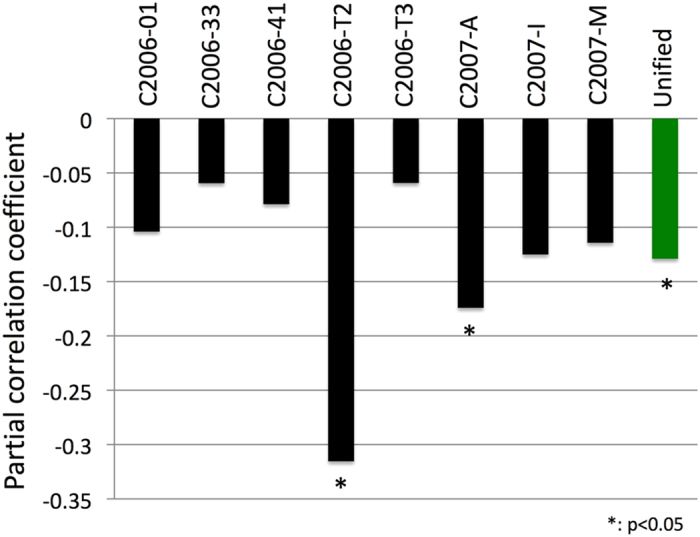
Observed partial correlation coefficients of the number of processed tasks between active and inactive workers among various colonies. Partial correlations in the number of processed tasks during an observation period between active (top 10%) and inactive (bottom 10%) workers controlling for the activity of the entire colony (intermediate 80%) in *Myrmica kotokui* colonies. All eight colonies show statistically significant negative correlations (black bars) between tasks processed by active and inactive workers. The average correlation over the colonies (green bar) is significantly negative, suggesting that task replacement occurred in actual colonies.

We tested whether the predicted correlation existed between the inactive and active workers. Because the activity of a worker is affected by the activities of the entire colony^29^, we used a partial correlation approach to control for the activity of the entire colony. The observed workers were divided into three classes based on their activities: (i) inactive (the lowest 10% in activity), (ii) active (the highest 10%) and (iii) intermediate (the middle 80%). For each colony, the partial correlation coefficient over all scans was calculated between the activities of the inactive workers and those of the active workers by controlling for those of the intermediate workers as an index of the activity of the entire colony. All of the partial correlation coefficients were negative (exact binomial test; number of negatives =8, number of trials =8, *p* = 0.0078) and ranged from −0.059 to −0.315 (Fig. 3). A meta-analysis was conducted to unify the coefficient over all 8 colonies, resulting in a significant value of −0.129 ± 0.089 (±95% confidence limit, the green bar in Fig. 3). This result clearly shows that inactive workers become active when the active workers are resting.

## Discussion

The proposed advantage of among-worker variation in threshold of response to task-related stimuli depends on the existence of critical tasks that cannot be halted at any moment. The nests of social insects are always threatened by bacterial and fungal infections. In termites, eggs are susceptible to bacterial infections and die quickly if attending workers are removed^28^. Termite workers lick eggs and place anti-bacterial substances on the eggs^28^. In ants, egg licking by workers has also been observed (164–168 pp. in ref. 30). Non-intermittent egg care appears to be a prerequisite for the persistence of a colony among soil-dwelling social insects.

Task replacement is also common among ant workers. When workers from a colony have been removed, tasks are performed by other physical or behavioral castes^24^,^31^,^32^,^33^. We examined the activities of the least active 10% of workers when the most active 10% of workers were active and when they were inactive. We found that the least active 10% of workers exhibited more activity when the most active 10% of workers were inactive than when they were active (Fig. 2a,b). This phenomenon can be explained by task replacement by inactive workers. Inactive workers perform few tasks because they have high thresholds for responding to task-related stimuli. These workers have no fatigue (power = 10) and are capable of performing several tasks at any time. In fact, a previous study^24^ has shown that a portion of inactive workers began to work when all of the active workers were removed from a colony. In a rare disastrous event, such replacement provides an effective means for the continual processing of critical tasks, resulting in the long-term persistence of a colony (Fig. 1b).

Task replacement is also found in the empirical data as the negative correlation between active and inactive workers (Fig. 3). We should note that this correlation is rather weak, because we have to use partial correlation to eliminate the effects of variable colony activity levels^14^. Other factors may also weaken this correlation. However, it is important to note that all eight colonies exhibit negative correlation, suggesting that inactive workers tend to work when active workers are resting.

There are two major hypotheses explaining task replacement: foraging for work^34^ and variable response thresholds^35^. As evidenced by the existence of inactive workers from empirical studies^24^, ant workers should have variable response thresholds. Variation in response thresholds has been shown to occur among several social insect species^24^,^25^,^26^,^27^. A previous study^24^ has suggested that *M. kotokui* also exhibits variation in response thresholds. Therefore, the inactive workers observed in this species are likely to originate from this variability. In contrast, the foraging-for-work hypothesis predicts that task replacement should enhance the overall performance of the colony in conducting crucial tasks principally because task replacement reduces the probability of colony extinction. In the current model, the threshold is treated as an absolute value. However, recent evidence suggests that the threshold is more akin to a probability of acting^27^. We believe that the qualitative outcomes are similar when the action is probabilistic. In the future, we plan to test the outcomes of the model by using probabilistic thresholds as opposed to absolute thresholds.

Division of labor and age polyethism are exhibited by several social insect species (298–354 pp. in ref. 30). These conditions may have a profound effect on the predictions of the model. However, the current model has two states (performing a task or not). Therefore, we could not compare the outcomes of the model with the empirical data even if data on polyethism were available in the empirical data. This perspective would be an interesting extension of the current model.

In this study, both the model and empirical data indicate that the variable threshold system permitted the continuous processing of crucial tasks (e.g., egg care). Several proximate mechanisms have been proposed for the variable threshold system^35^,^36^,^37^. We propose that the persistence of a colony is an ultimate (evolutionary) benefit for the maintenance of variable thresholds. Thus, the persistence of a colony is the critical measure of colony fitness. This principle is functionally equivalent to the concept of geometric mean fitness used to measure reproductive success under stochastic environments^38^,^39^. Geometric mean fitness is the maximization of long-term growth rate that reduces the probability of extinction. Similarly, the persistence of a colony is equivalent to avoiding colony extinction. However, the causal factors of the two fitness measures are different. Under the concept of geometric mean fitness, environmental or demographic stochasticity increases the probability of extinction^38^. These factors are external to the functional unit of adaptation, namely, individuals. In contrast, under our concept of colony persistence, the failure to perform critical tasks continuously is the factor that increases the probability of colony extinction. Thus, in contrast to the concept of geometric mean fitness, the factor increasing extinction probability is internal and the functional unit of adaptation is the colony. Additional studies are needed to examine “apparently suboptimal” phenomena (e.g., the extensive widespread occurrence of inactive workers) from the perspective of long-term persistence.

Our results demonstrate that ant colonies adopt a bet-hedging strategy against the unavoidable demographic stochasticity of task processing so that the colony can persist in the long run. Another common form of bet-hedging is the trade-off between growth and reproduction^38^,^39^ (e.g., the trade-off between colony growth and alate production^40^). Our current findings differ from these studies in two major ways. First, our study addresses worker adaptation from the perspective of long-term colony survival, irrespective of the trade-off between colony growth and reproduction. Second, current adaptation is opposed to demographic stochasticity within a colony and to environmental stochasticity. Bet hedging as an adaptation is not the sole explanation for inactive workers. Indeed, there are several other non-mutually exclusive explanations^21^ for the existence of inactive workers.

In typical colonies, there should be a great deal of variation in the total workload of a colony. Previous studies have shown that the removal of some active workers increases the activity level of the remaining active workers but does not affect the inactive workers^10^,^41^,^42^,^43^,^44^,^45^. Only when all of the active workers are removed will the remaining inactive workers begin to work^24^. These results indicate that inactive workers are not present to serve as daily or seasonal workload replacements but instead provide insurance against a catastrophic disaster in the event that all of the active workers are not able to engage in crucial tasks. In such a scenario, these active workers are likely to be incapable of working, owing to tremendous fatigue. Thus, our findings suggest that inactive workers may primarily function to safeguard against the risk of colony extinction that may occur very rarely (e.g., once in the lifetime of a colony if it were ever to occur).

Our results may have implications for the organization of societies in general. Specifically, a society without any reserves will be unable to persist over the long term. Natural selection may only “satisfice” short-term productivity instead of maximizing it (38–40 pp. in ref. 46)^47^. Therefore, all existing long-lasting societies should be adapted to long-term sustainability using bet hedging or preparing reservoirs of lazy workers.

## Methods

### Study organism and colony rearing

The study organism is *Myrmica kotokui*, a typical monomorphic ant^48^. This species forms colonies in fallen rotten wood/trunks and moves deep into the ground only during winter. The average colony size is approximately a few hundred individuals with a single queen. Details of field collection and rearing of colonies are described in a previous paper^24^. We collected five and three queenright colonies in May of 2006 and 2007, respectively, from the Tomakomai Experimental Forest of Hokkaido University in southwestern Hokkaido, Japan. All of the collected colonies contained a single queen, and workers ranged from 225 to 421 individuals. These collected colonies were reared in the laboratory for one month before experiments were conducted.

From each of these collected colonies, we established eight experimental colonies consisting of 150 workers, a queen, eggs and larvae housed in an artificial nest (plastic container with a plaster floor; 30 × 22 × 6 cm). In each plastic container, a single square chamber (10 × 8 × 1 cm) for a nest site was set at the center of the container. The chamber was covered with a clear glass plate with a thin red transparent cover so that individual markings on ants (see below) could be identified, and the ants behaved naturally. The nest space was connected to an adjacent foraging area by a 1-cm tunnel. We fed the colonies with commercially available insect food (Konchu-no-mitsu DXTM, Marukan, Osaka, Japan) ad libitum.

All workers in the experimental colonies were marked individually with Paint Markers^TM^ (Mitsubishi Inc., Tokyo, Japan). Ten colors and three marking points (between the eyes, pronotum and gaster) allowed us to discriminate among max. 1000 individuals. New workers emerging from pupae were marked within one day of emergence. All of the experimental colonies were reared in the laboratory for at least two weeks before beginning behavioral observations.

### Simulation

We randomly placed 75 workers on a lattice space with 2500 (50 × 50) cells on which tasks appeared randomly following a given probability (0.006 to 0.3) in each time step. A task has a stimulus (initial value = 5.001) at emergence. This stimulus increases by one value in the next step when the task is not processed. Each worker is assigned a threshold value. In the variable threshold system, the value is taken randomly from an integer between 0 and 10 following a normal distribution (mean = 5, binomial approximation). In addition, all of the workers are given the threshold value of 5 in the invariable system. In the next time step, a worker moves to any of adjacent four cells with a probability of 0.5. When another worker is already on the cell to which a worker moves, this movement is canceled. When the worker moves to a cell with a task, the worker will process the task if it has a higher stimulus than the worker’s threshold value.

An inactive worker becomes active due to the presence of a high stimulus. However, an inactive worker will return to inactivity immediately after this high-stimulus task has been processed because the threshold value of inactive workers is kept constant within a simulation.

We selected a cell randomly. We then processed all of the operations (move, task processing, task emergence) on this cell. Then, we placed a flag on the selected cell. Next, we selected a cell with no flag randomly. We repeated this procedure 2500 times. At this stage, all of the cells had been processed once. We then deleted all of the flags and performed the next time step. To set fatigue in the model, we set a variable “power” for each worker. The initial power was 10. When a worker processes a task, the worker’s power becomes zero. The worker cannot do anything until its power is back to 10. The worker’s power increases by a given recovery rate in each time step. We varied the degree of fatigue by altering this rate. We repeated this procedure for 1000 time steps per trial, and five trials were repeated for a set of the two parameters: (1) degree of fatigue and (2) task appearance rate. We recorded the following data in each time step and compared them between the cases with and without threshold variance: 1) the number of processed tasks and 2) the first step with no task processing. The colony was assumed to go extinct at this first blank step.

### Behavioral observations

We conducted observations on 8 colonies of the ant *Myrmica kotokui* using a scan-sampling method^49^. Once these behaviors had been defined through preliminary observations, scan data were collected over a 32-day period from late July to late August in 2006 and 2007. An observation cycle consisted of three continuous days of observations and one day without. A total of eight observation cycles were conducted during the observation period. To minimize the effect of diurnal changes in activity, each colony was scanned in the morning (9:00–10:00), early afternoon (13:00–14:00), and late afternoon (16:00–17:00). As a result, each individual ant was observed a total of nine times per observation cycle for a total of 72 scans (9 observations x 8 cycles) for each individual. However, because we were unable to view the identification marks of ants at the backside of the transparent cover of the nest chamber during any given scan, the total number of behavioral records was less than 72 for some individuals. We followed a previous study^24^ for the definitions and categorization of social behavior conducted by nest colony members. The categories of activities are allo-grooming, brood care, regurgitation and foraging. Categories of non-activities are rest, walking and self-grooming.

### Examination of task replacement by inactive workers

The average proportion of the resting behavior in a scanning was calculated for each colony. We calculated the activity (rate of social behaviors out of all behaviors) of each individual after defining each behavioral type (see above)^24^. Workers were divided into three classes following their activities in all the scans: inactive (the lowest 10%), active (the highest 10%) and intermediate (the middle 80%). If the inactive workers replace the tasks of the active workers, a negative correlation in the activity is expected between the highest and lowest classes. However, in a real colony, there is a cyclical change in colony activities^14^. When a colony is active, both active and inactive workers exhibit high activities. In contrast, when it is less active, they show low activities. By pooling these data, we get an apparent positive correlation between active and inactive workers even if there is no real positive correlation between them. In order to eliminate this apparent correlation, we used partial correlation to normalize the activity levels. Perform partial correlation requires an index that estimates the activity of the entire colony. This index cannot include data from the most active and least active workers to avoid tautological and double-counting problems. Therefore, the colony activity index was calculated from the activities of intermediate workers in a colony. There is no problem with using partial members of a colony for the determination of this index because we only use them to remove cyclical effects. Here, the activities of intermediate workers represent the real activities contributed to their colony (not including wandering/walking). Thus, we evaluated the partial correlation between the most active workers and the least active workers using the mid-active workers as an index of the overall activity of the entire colony. We used partial correlations for the empirical data and rank correlations for the simulation data. The partial correlation between active and inactive workers over the 72 scans (=[(3 observations/day) × 3 days + one resting day] × 8 times =3 × 3 × 8 samples) was calculated while controlling for the activity of the entire colony. Three samples taken in the same day were treated as independent samples. We used the activity of intermediate workers in a scanning as an index of the activity of the entire colony at that time. In this paper, “activities” are defined as behaviors that directly contribute to the health of other colony members. Therefore, all kinds of walking (including wandering) are excluded from activities (as the first approximation) since we find no direct colony benefits. Note that this definition is different from that used by Charbonneau and others^20^,^21^,^22^. For example, patrolling (a type of walking) for nursing needs is recognized as activity for mid-level workers specialized in nursing. Note that what definitions of activities are valid/better may depend on experiments, ant species, working clusters and various other conditions.

## Additional Information

**How to cite this article**: Hasegawa, E. *et al.* Lazy workers are necessary for long-term sustainability in insect societies. *Sci. Rep.* **6**, 20846; doi: 10.1038/srep20846 (2016).
